# Identification of Smad3‐related transcriptomes in type‐2 diabetic nephropathy by whole transcriptome RNA sequencing

**DOI:** 10.1111/jcmm.16133

**Published:** 2020-12-25

**Authors:** Qin Zhou, Honghong Guo, Chaolun Yu, Xiao‐Ru Huang, Liying Liang, Puhua Zhang, Jianwen Yu, Jizhou Zhang, Ting‐Fung Chan, Ronald C. W. Ma, Hui‐yao Lan

**Affiliations:** ^1^ Department of Nephrology The First Affiliated Hospital Sun Yat‐sen University Guangzhou China; ^2^ National Health Commission Key Laboratory of Nephrology The First Affiliated Hospital Sun Yat‐sen University Guangzhou China; ^3^ Guangdong Provincial Key Laboratory of Nephrology The First Affiliated Hospital Sun Yat‐sen University Guangzhou China; ^4^ State Key Laboratory for Biocontrol School of Life Sciences Sun Yat‐sen University Guangzhou China; ^5^ Department of Endocrinology Sun Yat‐sen Memorial Hospital Sun Yat‐sen University Guangzhou China; ^6^ Guangdong‐Hong Kong Joint Laboratory for Immunological and Genetic Kidney Disease Guangdong Academy of Medical Sciences Guangdong Provincial People’s Hospital Guangzhou China; ^7^ Department of Medicine and Therapeutics Li Ka Shing Institute of Health Sciences The Chinese University of Hong Kong Hong Kong China; ^8^ School of Life Sciences The Chinese University of Hong Kong Hong Kong China; ^9^ Guangdong‐Hong Kong Joint Laboratory on Immunological and Genetic Kidney Diseases The Chinese University of Hong Kong Hong Kong China

**Keywords:** alternative splicing, diabetic kidney disease, Smad3 deficiency, whole transcriptome

## Abstract

Smad3 deficiency prevents the development of type 2 diabetic nephropathy; however, the underlying molecular mechanisms remain unknown. In this study, we aimed to identify Smad3‐related genes involved in the pathogenesis of diabetic kidney disease. High‐throughput RNA sequencing was performed to profile the whole transcriptome in the diabetic kidney of Smad3 WT‐db/db, Smad3 KO‐db/db, Smad3^+/−^ db/db and their littermate control db/m mice at 20 weeks. The gene ontology, pathways and alternative splicing of differentially expressed protein‐coding genes and long non‐coding RNAs related to Smad3 in diabetic kidney were analysed. Compared to Smad3 WT‐db/db mice, Smad3 KO‐db/db mice exhibited an alteration of genes associated with RNA splicing and metabolism, whereas heterozygosity deletion of Smad3 (Smad3^+/−^ db/db mice) significantly altered genes related to cell division and cell cycle. Notably, three protein‐coding genes (Upk1b, Psca and Gdf15) and two lncRNAs (NONMMUG023520.2 and NONMMUG032975.2) were identified to be Smad3‐dependent and to be associated with the development of diabetic nephropathy. By using whole transcriptome RNA sequencing, we identified novel Smad3 transcripts related to the development of diabetic nephropathy. Thus, targeting these transcripts may represent a novel and effective therapy for diabetic nephropathy.

## 1 INTRODUCTION

The incidence of type 2 diabetes (T2D) has increased rapidly over recent decades and become a major public health crisis worldwide. Multiple factors, including obesity, ageing, diet, environment, genetic and epigenetic modifications, contribute to the development of diabetes. The increasing prevalence of T2D has led to the development of diabetic kidney disease (DKD),^1^ the most common cause of end‐stage renal disease. However, the precise mechanism of DKD in T2D remains unclear. There are several mouse models for studying T2D and DKD, such as mouse homozygous for the obese (Lep^ob^) and the diabetes (Lepr^db^) mutations. Studies have revealed that in BKS‐db/db (BKS.Cg‐ + Lepr^db^/+ Lepr^db^/J) mice, renal pathological changes are altered in the age of 12 weeks, which is accompanied with a markedly increase in high blood glucose and the development of urinary microalbumin excretion, indicating that kidney injury.^2^ Recent genetic and epigenetic studies using genome‐wide association study (GWAS) and epigenome‐wide association study have found that a number of genes are altered in DKD, including genomic DNA polymorphisms of collagen type IV alpha 3 chain (COL4A3, rs55703767),^3^ TGF‐β1( rs1800470), TNF‐α(rs1800629), IL‐6 (rs1800797) and IL‐1β (rs16944).^4^, ^5^, ^6^ However, functions of these genes associated with DKD remain unclear and need to be confirmed by further functional studies in experimental models of DKD.

TGF‐β1 has been implicated as an important factor that involves in various types of chronic kidney disease (CKD), including DKD.^7^ It regulates many aspects of cell function, including cell proliferation, accumulation of extra cellular matrix and apoptosis of renal cells in early diabetic kidneys. TGF‐β1 can be induced by many mediators of DKD, such as high glucose concentration, reactive oxygen species, advanced glycation end products, activated protein kinase C, renin‐angiotensin II‐aldosterone system components and endothelin,^8^, ^9^, ^10^ and are associated with the development of obesity and diabetes.^11^, ^12^ Blockade of TGF‐β by anti‐TGF‐β neutralization antibody can protect mice from diet‐induced obesity and diabetes.^11^ However, in a recent randomized, double‐blind, phase 2 clinical trial, the use of humanized TGF‐β1 monoclonal antibody (TGF‐β1 mAb) fails to show its therapeutic effect on patients with DKD.^13^ Findings from this clinical trial suggest the complexity of TGF‐β1 in DKD and general blockade of TGF‐β signalling may not be a good therapeutic approach for DKD due to the diverse role of TGF‐β in renal inflammation and fibrosis.^14^ Therefore, further studies to identify the downstream mediators of TGF‐β signalling that are directly and specifically regulate DKD are urgently needed. Our previous studies have found that Smad3 serves as a key mediator of TGF‐β signalling in several CKD models, including DKD.^14^, ^15^ By deleting Smad3 from db/db mice, we found that Smad3 knockout (KO)‐db/db mice were protected from DKD, with significantly reduced levels of albumin to creatinine, serum creatinine and histological injury in kidney, including mesangial matrix expansion and thickness of glomerular basement membrane over 12‐32 weeks.^16^ Interestingly, compared to Smad3 KO‐db/db, Smad3^+/−^ ‐db/db mice show no protection effect on DKD,^16^ revealing a necessary role for Smad3 in DKD. However, mechanisms through which Smad3 regulates DKD remain unknown, which was investigated in this study by performing a whole transcriptome RNA sequencing. A complete set of mRNA and non‐coding RNA (ncRNA) transcripts in Smad3 WT‐db/db, Smad3 KO‐db/db, heterozygous Smad3^+/−^‐db/db and their littermate controls Smad3 WT‐db/m and Smad3 KO‐db/m were compared.

## 2 MATERIALS AND METHODS

### 2.1 Animal models

Smad3^−/−^ (deletion of exon 8 and disruption of exon 7) on C57BL/6J (H‐2b) background (both sexes, aged 6‐8 weeks, 20‐25 g) were kindly provided by Dr Chunxia Deng.^17^ In briefly, C57BL/6J Smad3^+/−^ mice were intercrossed with C57BLKs/J Lepr^+/−^ (db/m) mice on a background to produce Smad3^+/−^ db/m heterozygous mice as previously described.^16^ Double‐heterozygous Smad3^+/−^ db/m male and female were intercrossed to generate double mutation of Smad3 and Lepr (Smad3 KO‐ db/db) and other genotype mice (Smad3 WT‐db/m; Smad3 KO‐db/m; Smad3 WT‐db/db; Smad3^+/−^ db/db). All animal husbandry and animal experiments were approved by the Animal Ethics Experimental Committee at the Chinese University of Hong Kong and confirmed to be in accordance with local regulations. Mice kidneys were collected at 20 weeks of age from mice with the five different genotypes.

### 2.2 RNA sequencing

Total RNA of kidney tissue was extracted from mice with five different genotypes (Smad3 WT‐db/m; Smad3 WT‐db/db; Smad3 KO‐db/m; Smad3 KO‐db/db; and Smad3^+/−^ db/db) at 20 weeks (n = 3‐5 for each genotype) with TRIzol reagent (Invitrogen) in accordance with the manufacturer's instructions. Two microgram of total RNA was used as starting material for ribosomal RNA (rRNA) depletion. rRNA depletion was performed by using Ribo‐Zero Gold rRNA Removal Kit (Human/Mouse/Rat), and cDNA libraries were prepared by KAPA Stranded RNA‐Seq Kit. Paired‐end sequencing was conducted on an Illumina HiSeq 1500 platform (Pair‐End sequencing of 101 bp) by The University of Hong Kong. The animal ID and grouping information were listed in Table S1.

### 2.3 Analysis of RNA‐Seq data

Using software from Illumina (bcl2fastq), sequencing reads were assigned into individual samples with each sample having an average throughput of 5.0 Gb (Table S2) and a total throughput of 99.9 Gb. In terms of sequence quality, an average of 94% of the bases achieved a quality score of Q30 where Q30 denotes the accuracy of a base call to be 99.9%.

In order to obtain high‐quality clean sequencing data, Fastp (version 0.19.4) was used to filter low‐quality reads, cut adapters and quality control raw FASTQ files to obtain clean reads.^18^ The clean reads of each sample were aligned against the mouse genome (UCSC/mm10) with HISAT2 (version 2.1.0)^19^ and then subsequently assembled by StringTie (version 1.3.4d)^20^ separately. All assemblies were merged into one transcriptome by TACO (Version 4.7).^21^ The newly assembled transcriptome was annotated with Gencode vM18 and NONCODE v5 using GffCompare (http://github.com/gpertea/gffcompare, version 0.10.1) to find novel transcripts. The distances between novel transcripts and reference protein‐coding transcripts were calculated by bedtools^22^ CPAT^23^ and PLEK (version 1.2)^24^ were used to calculate the coding potential of novel transcripts.

The abundance of the RNAs was quantified and normalized using Salmon (version 1.11.2).^25^ The Pearson correlation coefficients of transcriptome expression level were calculated based on normalized reads count. Tximport (version 1.12.3)^26^ was applied to import quantification of transcript expression into R. Then, differentially expressed genes were determined and visualized by edgeR and ggplot2, respectively. The threshold to determinate differential expression was FDR value <0.05 and |log_2_ (fold change) |> 1(down‐regulated or up‐regulated over twofold).

The bioinformatics analysis of RNA‐seq data was divided into four comparisons: (a) comparison of DEGs related to DKD in WT models (Smad3 WT‐db/db vs Smad3 WT‐db/m), (b) comparison of DEGs related to DKD in Smad3 KO models (Smad3 KO‐db/db vs Smad3 KO‐db/m), (c) comparison of DEGs related to Smad3 homozygous KO in DKD models (Smad3 KO‐db/db vs Smad3 WT‐db/db) and (d) comparison of DEGs related to Smad3 heterozygous deletion in DKD models (Smad3^+/−^ db/db vs Smad3 WT‐db/db).

### 2.4 Pathway and ontology analysis of differentially expressed genes

The profile of GO enrichment and Kyoto Encyclopedia of Genes and Genomes (KEGG) pathways analysis performed by R package ClusterProfiler^27^ was used to predict potential functional roles for significant differentially expressed protein‐coding genes and lncRNAs; the cut‐off threshold was defined as *P*‐adjust < .05.

The biological function of the uncharacterized lncRNAs can be predicted based on the functionality of the nearby protein‐coding genes (lncRNA cis‐genes). We searched for the protein‐coding genes closest to all the identified lncRNAs by calculating the distance between the two chromosomal position by using the closest function in bedtools software.^22^ GO and KEGG enrichments with *P*‐adjust < .05 were considered significantly enriched by using R package ClusterProfiler.^27^

### 2.5 Detection and quantification of alternative splicing variants

The tool rMATS was used to analyse the alternative splicing (AS) profiles and evaluates the mRNA splicing patterns. Five types of AS events were quantified in each comparison. AS events are including SE: Skipped exon, MXE: Mutually exclusive exon, A5SS: Alternative 5′ splice site, A3SS: Alternative 3′ splice site and RI: Retained intron.^28^

### 2.6 The co‐expression network of lncRNA and protein‐coding RNA

Pearson correlation coefficient (pcc) of differentially expressed lncRNA and mRNA was calculated by R (version 3.5.1, R Core Team 2018, https://www.R‐project.org/.) The corresponding lncRNA and protein‐coding gene pair were calculated at the cut‐off value at pcc > 0.9 and *P*‐value < .05 in comparisons 1 and 4, pcc > 0.99 and *P*‐value < .05 in comparisons 2 and 3 were considered as significant, respectively. Cytoscape (v 3.7.1) was used to draw the co‐expression network map of lncRNA and protein‐coding gene.

### 2.7 Quantitative real‐time PCR (qPCR)

RNA from the kidney tissues of mice (n = 5‐7 for each group, respectively) was reverse transcribed to cDNA by using PrimeScript RT Reagent Kit with gDNA Eraser (Takara). Gene expression was quantified by real‐time PCR with TB green Kit (Takara). Quantitative real‐time PCR was carried out on ABI 7900 system using the following program: 95°C for 1 minute; 40 cycles of 95°C for 15 seconds, 58°C for 15 seconds, and 72°C for 30 seconds and dissociation steps. Primers for all the tested DEGs and β‐actin, an endogenous control for normalization, are listed in Table S3. The relative expression of target genes to β‐actin was calculated by using $2^{‐\Delta \Delta C_{t}}$ method.^29^ Data were shown as mean ± SEM. Statistical analysis was performed by using one‐way ANOVA followed by Newman‐Keuls multiple comparison test from GraphPad Prism 5.0 (Graph Pad Software).

## 3 RESULTS

### 3.1 Overview analysis of transcriptomic RNA‐seq

The earliest clinical sign of kidney damage from diabetes is the presence of albuminuria. According to the guidelines of National Kidney Foundation‐Kidney Disease Outcomes Quality Initiative (NKF KDOQI), DKD was divided into microalbuminuria (albumin/creatinine ratio, ACR 30‐300 mg/g) and macroalbuminuria (ACR > 300 mg/g) based on the level of albuminuria. As shown in our previous study, kidney injury in the db/db was evidenced by the development of urinary microalbumin (ACR), which was increased gradually from 12 to 20 weeks, peaked over 20‐32 weeks.^16^ A whole transcriptomic RNA‐seq analysis of kidney tissue from five different groups (Smad3 WT‐db/m; Smad3 WT‐db/db; Smad3 KO‐db/m; Smad3 KO‐db/db; and Smad3 ^+/−^ db/db) at 20 weeks was performed.

**Figure 1:**
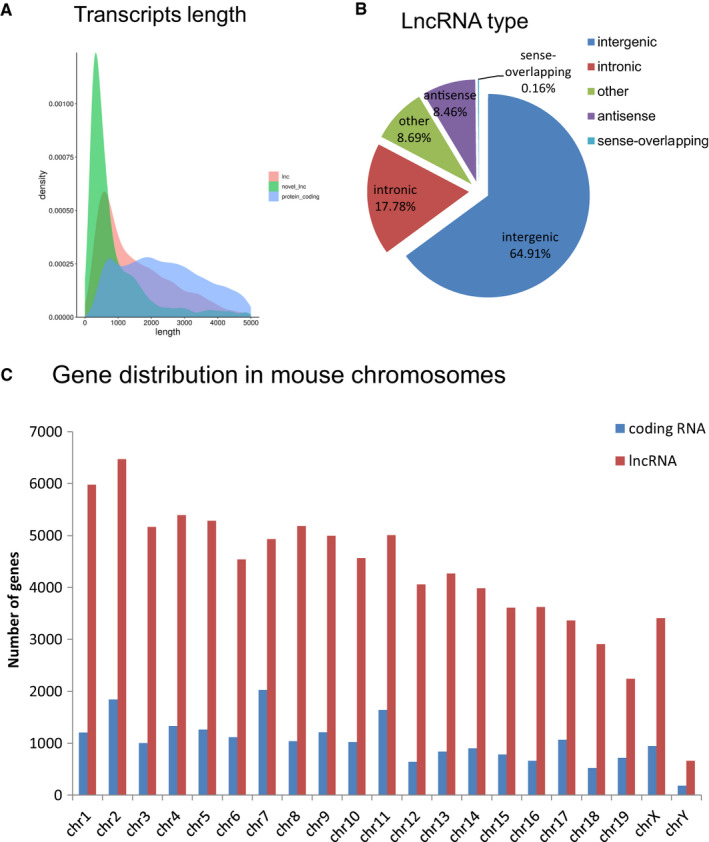
Overview analysis of transcriptomic RNA‐seq. A, The length of all lncRNAs, protein‐coding RNA and predict novel lncRNAs. B, The classification of lncRNAs. C, The distribution of protein‐coding and lncRNAs in all the mouse chromosomes

The transcripts obtained by RNA‐Seq were annotated with Gencode vM18 and NONCODEv5. The TPM value was used to quantify the assembled transcriptome, including differentially expressed protein‐coding and lncRNA genes (Table S4). The length of most transcripts was concentrated within 1000 nt (Figure 1A). The transcripts with length over 200 nt and more than two exon without coding potential which calculated by CPAT and PLEK were selected as lncRNAs for further analysis.^30^, ^31^ These lncRNAs were classified into five categories according to their genomic loci: antisense, sense‐overlapping, intergenic, intronic and other. The distribution proportion of lncRNAs is schematically presented in a pie chart (Figure 1B). The majority of the identified lncRNAs were intergenic (64.91%). Column chart in Figure 1C showed these protein‐coding and lncRNAs were widely distributed in all the mouse chromosomes. The total number of novel lncRNA was 1073, which was found based on GffCompare, and the detailed information of them (including genomic location, type and closest protein‐coding gene) was listed in Table S5. Pearson correlation analysis showed that all the transcripts (including protein‐coding RNA and lncRNA) between these 20 samples were highly correlated (*r* = .90‐.97, *P* < .01; Figure S1).

### 3.2 Biological function analysis of altered protein‐coding genes in db mouse kidney with or without Smad3 knockout

Compared to control mice, further analysis revealed that many protein‐coding genes were altered in the diabetic kidney of Smad3 WT or Smad3 KO db/db mice at 20 weeks. The details of these protein‐coding genes (including Gene ID, Gene name, logFC, *P*‐value, FDR) in each comparison were listed in Table S6. As summarized in Table 1, compared to Smad3 WT‐db/m control, expression of 29 genes increased over onefold in Smad3 WT‐db/db kidney, while that of 89 genes decreased over onefold, indicating that more protein‐coding genes were inhibited in diabetic kidney. In Smad3 KO‐db/db model, the number of up‐ and down‐regulated genes was 272 and 236 when compared to Smad3 KO‐ db/m, while there were 794 and 182 when compared to Smad3 WT‐db/db. In Smad3^+/−^ db/db, the number of altered genes was much less, only 117 up‐regulated and 11 down‐regulated, respectively, when compared to Smad3 WT‐db/db. The data implicated that Smad3 is a strong gene inhibitor in the kidney of db/db mice and the effect of homozygous KO of Smad3 gene is more profound than heterozygous in db/db mouse kidney.

**Table 1:** The number of significant differentially expressed protein‐coding genes (PCG) and lncRNAs in the kidney of control and db mice

Comparison	Groups		Total	Up‐regulated	Down‐regulated
1	Smad3 WT‐db/db vs Smad3 WT‐db/m	PCG	118	29	89
		LncRNA	45	12	33
2	Smad3 KO‐db/db vs Smad3 KO‐db/m	PCG	508	272	236
		LncRNA	117	42	75
3	Smad3 KO‐db/db vs Smad3 WT‐db/db	PCG	976	794	182
		LncRNA	505	331	174
4	Smad3^+/−^ db/db vs Smad3 WT‐db/db	PCG	128	117	11
		LncRNA	29	17	12

Note

Threshold: |logFC|>1 and FDR < 0.05.

As shown in Table 2, in Smad3 WT‐db/db, Crabp1, Lpo, Igfbp2, Spon2 and Upk1b were the most up‐regulated genes while Mup19, Ucp1, Akr1c18, Mup3 and Il11ra2 were the most decreased genes when compared to Smad3 WT‐db/m. Not surprisingly, metabolism and immune‐related genes were strongly affected. Crabp1 is the primary mediator of the noncanonical activities of all‐trans retinoic acid in cancer, the latter is the principle active metabolite of vitamin A.^32^ Lactoperoxidase is an oxidoreductase which widely distributed in mammalian tissues and secretions, and has important function of natural antimicrobial defence.^33^ Mup19 and Mup3 are small proteins belong to the lipocalin superfamily, which have the ability to bind small organic molecules such as pheromones.^34^ They play key roles in energy metabolism and potentially contribute to the development of metabolic diseases such as T2D. It is reported that Mup19 is regulated by lipid metabolism and also play an important role in lipid metabolism and deposition in kidney.^35^ Ucp1 functions to uncouple mitochondrial fatty acid oxidation from the production of ATP.^36^ Akr1c18 (Aldo‐keto reductase 1c18) belong to the AKR superfamily, act as NADP(H)‐dependent oxidoreductases with potential roles in the regulation of steroid metabolism.^37^

**Table 2:** Top 10 significantly changed protein‐coding genes (PCG) and lncRNAs in each comparison

Comparison	Group	Up‐regulated genes	Down‐regulated genes
1	Smad3 WT‐db/db vs Smad3 WT‐db/m	Crabp1 (cellular retinoic acid binding protein I) Lpo (lactoperoxidase) Igfbp2 (insulin‐like growth factor binding protein 2) Spon2 (spondin 2) Upk1b (uroplakin 1B) Lnc‐Lpin2 (NONMMUG018828.2) Lnc‐Tspan2(NONMMUG027474.2) Lnc‐Cdc73(NONMMUG001953.2) Lnc‐Zer1(NONMMUG022493.2) Lnc‐Abcg2(NONMMUG035489.2)	Mup19 (major urinary protein 19) Ucp1 (uncoupling protein 1) Akr1c18 (aldo‐keto reductase family 1, member C18) Mup3 (major urinary protein 3) Il11ra2 (interleukin‐11 receptor subunit alpha‐2‐like) Lnc‐Nus1 (NONMMUG003616.2) Lnc‐Sptssa (NONMMUG009013.2) Lnc‐Zbtb44(NONMMUG042380.2 Lnc‐Exoc3l2(NONMMUG094809.1) Lnc‐Pxdn (NONMMUG008728.2)
2	Smad3 KO‐db/db vs Smad3 KO‐ db/m	Ugt1a8 (UDP glucuronosyltransferase 1 family, polypeptide A8) Ccdc7a (coiled‐coil domain containing 7A) Rsph14 (radial spoke head homolog 14) Higd1c (HIG1 domain family, member 1C) Grm5 (glutamate receptor, metabotropic 5) Lnc‐Slc5a12 (NONMMUG024138.2) Lnc‐Ubxn7 (NONMMUG016266.2) Lnc‐Cebpzos (NONMMUG018930.2) Lnc‐Kynu (NONMMUG068163.1) Lnc‐Gnpnat1 (NONMMUG012781.2)	Eno1b (enolase 1B, retrotransposed) Akr1c18 (aldo‐keto reductase family 1, member C18) Ucp1 (uncoupling protein 1) Cox8b (cytochrome c oxidase subunit 8B) Hist1h2bq (histone cluster 1, H2bq) Lnc‐Runx1t1 (NONMMUG028559.2) Lnc‐Met (NONMMUG034685.2) Lnc‐Nacc1 (NONMMUG041098.2) Lnc‐Ptprd (NONMMUG029591.2) Lnc‐Tanc1 (NONMMUG023099.2)
3	Smad3 KO‐db/db vs Smad3 WT‐db/db	Olfr1321 (olfactory receptor 1150, pseudogene 1) Olfr643 (olfactory receptor 643) Vmn2r100 (vomeronasal 2, receptor 100) Vmn1r173 (vomeronasal 1 receptor 173) Il11ra2 (interleukin‐11 receptor subunit alpha‐2‐like) Lnc‐Nus1(NONMMUG003616.2) Lnc‐Slc5a12 (NONMMUG024138.2) Lnc‐Cebpzos (NONMMUG018930.2) Lnc‐Spag9 (NONMMUG007310.2) Lnc‐Gsr (NONMMUG040267.2)	Hist1h2bq (histone cluster 1, H2bq) Eno1b (enolase 1B, retrotransposed) Lep (leptin) Slco1a1 (solute carrier organic anion transporter family, member 1a1) Ivl (involucrin) Lnc‐Met (NONMMUG034685.2) Lnc‐Trps1 (NONMMUG014490.2) Lnc‐Ptprd (NONMMUG029591.2) Lnc‐Nacc1 (NONMMUG041098.2) Lnc‐Zmym2 (NONMMUG090129.1)
4	Smad3^+/−^db/db vs Smad3 WT‐db/db	Mup19 (major urinary protein 19) Cyp2c37(cytochrome P450, family 2. subfamily c, polypeptide 37) Cyp3a41a (cytochrome P450, family 3, subfamily a, polypeptide 41A) Apof (apolipoprotein F) Ngp (neutrophilic granule protein) Lnc‐Nus1(NONMMUG003616.2) Lnc‐Pitpna(NONMMUG053244.1) Lnc‐Eif4ebp2(NONMMUG003753.2) Lnc‐Sh3bgrl2(NONMMUG043499.2) Lnc‐Sptssa(NONMMUG009013.2)	Nxph1 (neurexophilin 1) Sult2a3 (sulfotransferase family 2A, dehydroepiandrosterone (DHEA)‐preferring, member 3) Pde11a (phosphodiesterase 11A) Ddit4 (DNA‐damage‐inducible transcript 4) Tsku (tsukushi, small leucine rich proteoglycan) Lnc‐Tspan2(NONMMUG027474.2) Lnc‐Gls(NONMMUG000589.2) Lnc‐Rab8b(NONMMUG043160.2) Lnc‐Zfp942(NONMMUG017656.2) Lnc‐Mtx3(NONMMUG011595.2)

Note

Threshold: FDR < 0.05, Predict genes were not included.

In Smad3 KO‐db/db, Ugt1a8, Ccdc7a, Rsph14, Higd1c and Grm5 were the most up‐regulated protein‐coding genes when compared to Smad3 KO‐dm, whereas Eno1b, Akr1c18, Ucp1, Cox8b and Hist1h2bq were greatly decreased. The alteration of metabolic related gene is also very significant. Ugt1a8 (UDP glucuronosyltransferase 1 family, polypeptide A8), belonged to UGT superfamily, can efficiently use UDP‐glucuronic acid as the glycosyl donor and are invaluable in detoxifying both endogenous and foreign chemicals.^38^ Eno1b is a pseudogene of the alpha‐enolase, the latter ubiquitous expressed in multiple tissues and most abundant in kidney adult. The core ATP produce and thermo‐control gene Ucp1 as well as mitochondrial function gene Cox8b is related to the obesity and diabetes.^39^ Akr1c18 and Ucp1 are decreased dramatically in both Smad3 WT and Smad3 KO‐ db/db, indicating they are critical in diabetes‐related renal injury.

When compared to Smad3 WT‐db/db, deletion of Smad3 KO from db/db mice up‐regulated renal Olfr1321, Olfr643, Vmn2r100, Vmn1r173 and Il11ra2 expression but decreased the expression of Hist1h2bq, Eno1b, Lep, Slco1a1 and Ivl. The up‐regulation of olfactory receptor (Olfr) and vomeronasal receptor genes was very noticeable.^40^ Both genes are the large protein‐coding gene families in response to the innate immunity as well as social and sexual behaviours in mice.^41^, ^42^ Hist1h2bq belongs to histone cluster 1 H2B family, which is conserved in human, chimpanzee, dog, cow and mouse. It is most highly expressed in thymus and spleen, indicating it may critical in the mature of immune cell. Leptin has a critical role as a hormone and a cytokine in regulating metabolism and pro‐inflammatory responses. Decreased leptin concentrations in serum in obese patients significantly protect those patients from developing insulin resistance, T2D, cardiovascular and autoimmune diseases.^43^ Solute carrier organic anion transporter family member (Slco) 1a1 is responsible for serum metabolites of bile acids and can be down‐regulated by tumour necrosis factor‐α and transforming growth factor‐β1.^44^

In Smad3 heterozygous KO (Smad3^+/−^ db/db), Mup19, Cyp2c37, Cyp3a41a, Apof and Ngp were the most up‐regulated genes while Nxph1, Sult2a3, Pde11a, Ddit4 and Tsku were the most down‐regulated genes when compared to Smad3 WT‐db/db. The Mup19 level up‐regulated both in the kidney of Smad3 WT‐db/db and Smad3^+/−^ db/db mice. Metabolic genes (Cytochrome P‐450 superfamily members Cyp2c37, Cyp3a41a) and lipoprotein modulating gene (Apolipoprotein F) were also raised in Smad3^+/−^ db/db. Nxph1 loci were found associated with the plasma triglyceride (TG) response to ω‐3 fatty acid supplementation in a GWAS.^45^ Although the profile of DEG in Smad3 heterozygous KO diabetic mice is quite different from Smad3 homozygous KO, the DEGs in two groups show protective roles in diabetic renal injury.

**Figure 2:**
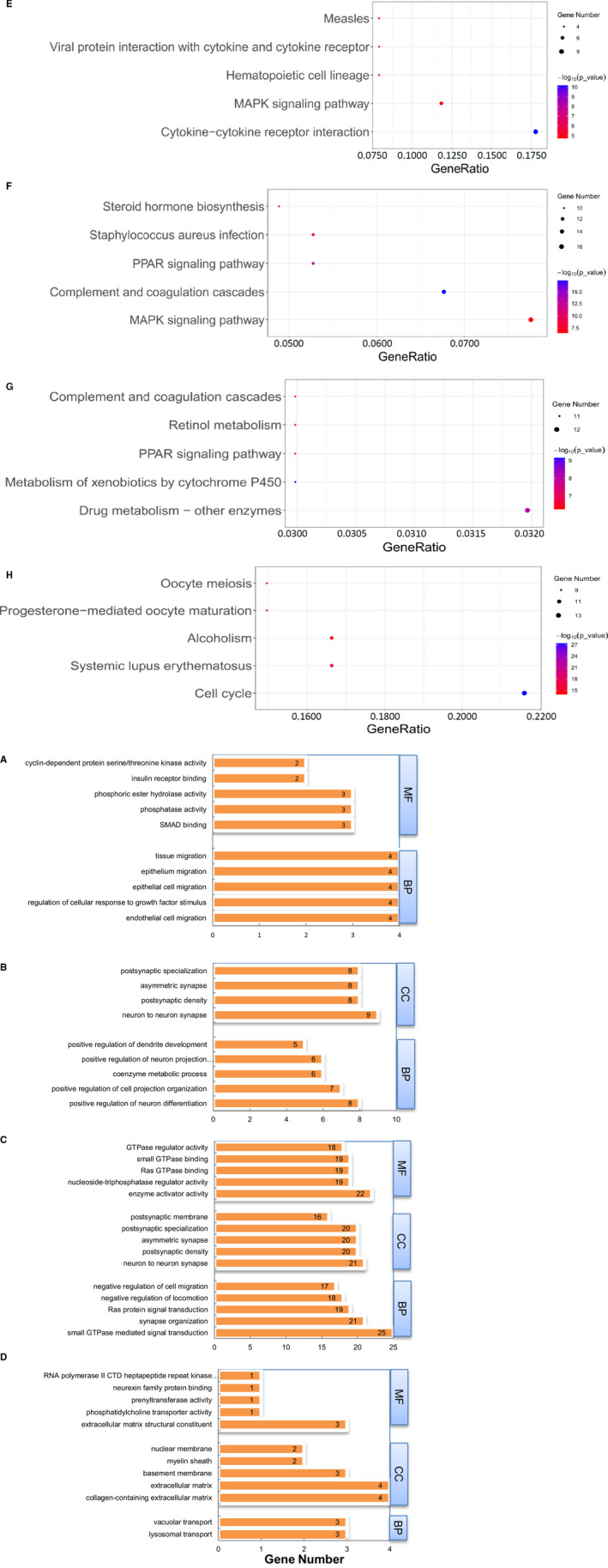
The top five significant GO terms (A‐D) and KEGG pathways (E‐H) of differentially expressed protein‐coding genes in comparisons 1‐4. BP, biological processes; CC, cellular component; MF, molecular function. When the number of significant GO terms and KEGG pathways was <5, actual number was listed in the figure

To further verify the biological functional of DEGs in db/db model, DEGs were selected for GO and KEGG analysis. Gene Ontology describes genes in three non‐overlapping domains which including biological processes (BP), cellular component (CC) and molecular function (MF). We summarized the number and detail information of significant GO terms in Table S7 and S8. The top five most significant GO terms of down‐regulated or up‐regulated genes in four comparisons were shown in Figure 2.

In Figure 2A (Smad3 WT‐db/db vs Smad3 WT‐db/m), most of the DEGs in db model, either up‐regulated or down‐regulated, were significantly enriched in metabolically related biological process. Up‐regulated DEGs were enriched in GO_BP of hormone metabolic process, in response to metal ion, ERK1 and ERK2 cascade, small molecule catabolic process, and peptide hormone. The down‐regulated DEGs were enriched in those with regulatory role in protein secretion, fatty acid metabolic process, T cell activation, multicellular organismal homeostasis and organic hydroxy compound metabolic process. As shown in Figure 2B, compared to Smad3 KO‐db/m, DEGs up‐regulated in Smad3 KO‐db/db were significantly enriched in RNA splicing and xenobiotic metabolism related biological process. The most significant GO_BP in down‐regulated DEGs including those in response to topologically incorrect protein, angiogenesis, protein kinase activity, reactive oxygen species and leucocyte cell‐cell adhesion. The results of GO analysis showed that Smad3 inhibited genes related to RNA splicing and xenobiotic metabolism in diabetic kidney, indicating that Smad3 may participate in alternatively splicing. When compared to Smad3WT‐db/db as shown in Figure 2C, the most significant GO_BP of up‐regulated DEGs in Smad3 KO‐db/db was related to RNA splicing, response to pheromone, cellular response to interferon‐beta, response to interferon‐beta, histone H3‐K4 methylation. Down‐regulated DEGs in Smad3 KO‐db/db were enriched in metabolic processes, such as organic hydroxy compound metabolic process, alcohol metabolic process, fatty acid metabolic process, organic anion transport and carboxylic acid transport. It is possible to infer that homozygous deletion of Smad3 may have essential impacts on metabolism and promote the expression of genes related to RNA splicing in diabetic kidney. It is worth noting that in Figure 2D, 31 up‐regulated DEGs (31/117) in Smad3^+/−^ db/db were significantly enriched in GO_BP of nuclear division and GO_CC of spindle (28/117).

To further identify the function of DEGs in each group, pathway analysis of the protein‐coding genes was performed by using ClusterProfiler. The significant pathways in each comparison were summarized in Table S9. The top five most significant KEGG pathway of DEGs in four comparisons was shown in Figure 2E‐H. Enrichment analysis revealed that DEGs in Smad3 WT‐db/db were mainly in those pathways related to cytokine‐cytokine receptor interaction and MAPK (Mitogen‐activated protein kinases) when compared to their WT db/m (Figure 2E). In comparison 2 (Figure 2F), DEGs in Smad3^−/−^ db/db were significantly clustered in the pathways related to MAPK, complement and coagulation cascades, peroxisome proliferators‐activated receptors (PPAR), staphylococcus aureus infection and steroid hormone biosynthesis. Genes participating in MAPK signalling pathway were dramatically inhibited in diabetic models, either in Smad3 WT or Smad3 KO db/db mouse models. In comparison 3 (Figure 2G), Smad3 deficiency affected the genes expression in the signalling pathway of drug metabolism‐other enzymes, metabolism of xenobiotics by cytochrome P450, PPAR, retinol metabolism, complement and coagulation cascades. In comparison 4 (Figure 2H), Smad3^+/−^ in db/db mainly increase the genes in the pathway of cell cycle, systemic lupus erythematosus, alcoholism, progesterone‐mediated oocyte maturation and oocyte meiosis.

The results of GO and KEGG indicated that genes engaged in metabolism, inflammation and immunity were dysregulated in either Smad3 WT or Smad3 KO diabetic kidneys of db/db mice. Smad3 homozygous and heterozygous deletion had different effects on kidney function during the process of diabetic kidney injury. Smad3 homozygous KO significantly affected the genes which have function in metabolism and RNA splicing process, whereas Smad3 heterozygous deletion has more effects on the nuclear division and cell cycle. The bioinformatics data are in accordance with our previous observation that Smad3 homozygous deletion rather than Smad3 heterozygosity in db/db mice had significant effect on protecting against renal dysfunction and histological lesions.^11^

### 3.3 Biological function analysis of LncRNA in db kidney with or without Smad3 knockout

High‐throughput sequencing revealed that the majority of the human or mouse transcriptome can be referred as ncRNA. LncRNAs are a class of ncRNAs mainly uncharacterized and have unknown biological functions. Compared to control, many lncRNAs were also altered in Smad3 WT‐db/db or Smad3 KO‐db/db mouse models. The number of significantly changed lncRNAs was summarized in Table 1, and the details of these lncRNAs (including Gene ID, Fold Change, TPM, *P*‐value, FDR and closest protein‐coding gene) were listed in Table S10.

Although the total number of ncRNAs identified by RNA‐seq was larger than protein‐coding gene, significantly changed lncRNAs was less than protein‐coding genes in each comparison. The top five most up‐ or down‐regulated lncRNAs and their closest protein‐coding genes in each comparison were list in Table 2. Many researchers observed that lncRNA expression is often correlated with the expression of nearby genes.^46^ As shown in Table 2, compared to Smad3 WT‐db/m, Lpin2, Tspan2, Cdc73, Zer1 and Abcg2 were the top five up‐regulated while Nus1, Sptssa, Zbtb44, Exoc3l2 and Pxdn were the most decreased lncRNA‐closest genes in Smad3 WT‐db/db.

**Figure 3:**
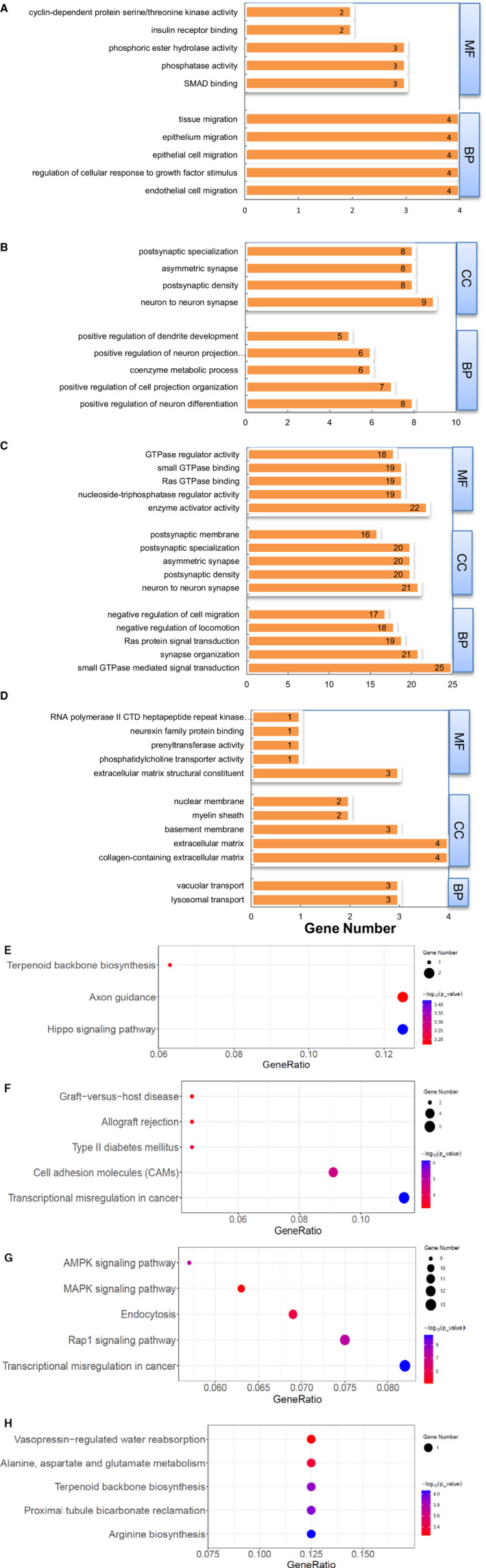
The top five significant GO terms (A‐D) and KEGG pathways (E‐H) of differentially expressed lncRNAs in comparisons 1‐4. BP, biological processes; MF, molecular function; CC, cellular component. When the number of GO terms and KEGG pathways was less than 5, actual number was listed in the figure

Then, we also predicted the potential functional role of differentially expressed lncRNAs by analysing their closest genes in GO and KEGG database. The number of significant GO terms of differentially expressed lncRNAs was summarized in Table S11. The altered lncRNAs in each comparison were enriched in certain biological functions, respectively (Table S12). As shown in Figure 3A, in comparison 1, lncRNA‐closest genes in Smad3 WT‐db/db were enriched in GO_BP of endothelial cell migration and GO_MF of “SMAD binding”. In Figure 3B, the most significant GO_BP of “positive regulation of neuron differentiation” was found in differentially expressed lncRNAs in Smad3 KO‐db/db compared to Smad3 KO‐db/m. Compared to Smad3 WT‐db/db, up‐regulated lncRNAs in Smad3 KO‐db/db were clustered in GO_BP of small GTPase mediated signal transduction (Figure 3C). In Smad3^+/−^db/db vs Smad3 WT‐db/db, dysregulated lncRNAs were enriched in GO_BP of lysosomal and vacuolar transport (Figure 3D).

KEGG pathway analysis of lncRNA‐closest gene was performed to predict the potential functional role of altered lncRNAs (Table S13). As shown in Figure 3E, lncRNA‐closest protein‐coding genes were enriched in the pathways of “Hippo signaling pathway”, “Axon guidance” and “Terpenoid backbone biosynthesis” in Smad3 WT‐db/db when compared to Smad3 WT‐db/m. In Smad3 KO‐db/db, these genes were significantly enriched in the pathways of “Transcriptional misregulation in cancer”, “Cell adhesion molecules” and “Type II diabetes mellitus” when compared to Smad3 KO‐db/m (Figure 3F), whereas “Transcriptional misregulation in cancer”, “Rap1 signaling pathway”, “Endocytosis”, “MAPK signaling pathway” and “AMPK signaling pathway” were the most significant pathways compared to Smad3 WT‐db/db (Figure 3G). In Smad3^+/−^db/db, due to the small number of differential expressed lncRNAs, only one lncRNA‐closest gene was significantly clustered in several pathways (Figure 3H).

### 3.4 Smad3 related DEGs in db/db mouse kidneys

To identify Smad3‐regulated genes in db/db models, DEGs with differential expression direction in comparisons 1 and 2 were considered as genes related to diabetic kidney injury and regulated by Smad3. As shown in Table 3, three protein‐coding genes (Upk1b, Psca and Gdf15) and two lncRNAs (NONMMUG023520.2 and NONMMUG032975.2) were found expressed diversely in comparisons 1 and 2. According to previous publication, these genes or lncRNA‐closest genes are critical in diabetes or renal function. Upk1b (Uroplakin 1b) is a four transmembrane protein, and critical in urinary tract development and urothelial differentiation and homeostasis.^47^ Gdf15 (growth/differentiation factor 15) is a distant member of the bone morphogenetic protein subfamily of the transforming growth factor‐beta, currently being investigated as a potential target for the treatment of obesity and type 1 diabetes mellitus.^48^ It is weakly expressed under normal conditions, but it is sharply up‐regulated during inflammation and acts as an autocrine regulator of macrophage activation.^49^ G3bp2 (Ras GTPase‐activating protein SH3 domain‐binding protein 2) is a multifunctional RNA‐binding protein involved in stress granule assembly, increased in the renal of DKD.^50^ However, more experimental evident was needed to prove whether these DEGs were regulated by Smad3 in the diabetic kidney.

**Table 3:** Differentially expressed genes which have different expression direction in comparisons 1 and 2

Gene ID	Gene (or closet PCG) Name	Comparison 1		Comparison 2	
		LogFC	FDR	LogFC	FDR
ENSMUSG00000049436.5	Upk1b	2.34	0.0083	−2.06	0.0284
ENSMUSG00000022598.6	Psca	1.98	0.0399	−2.32	0.0101
ENSMUSG00000038508.7	Gdf15	1.17	0.0331	−1.69	0.0390
NONMMUG023520.2	Sestd1	6.95	0.0239	−8.26	0.0047
NONMMUG032975.2	G3bp2	−7.31	0.0065	7.86	0.0041

### 3.5 Alternative splicing of mRNA

**Figure 4:**
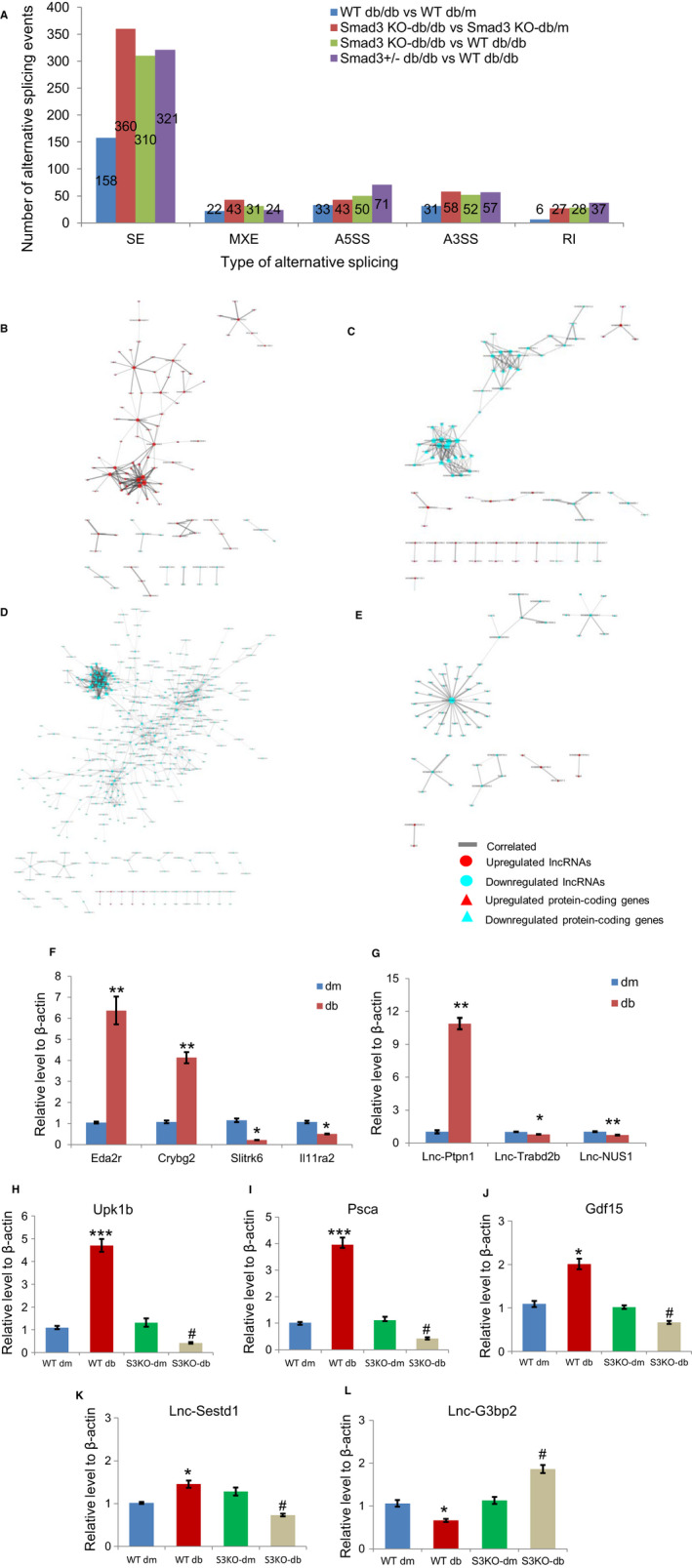
A, The number of alternative splicing events in each comparison. A3SS, Alternative 3′ splice site; A5SS, Alternative 5′ splice site; MXE, Mutually exclusive exon; RI, Retained intron; SE, Skipped exon. B‐E, The co‐expression network of lncRNAs and protein‐coding genes in comparisons 1‐4. The relative expression levels of protein‐coding (F) and lncRNA genes (G) in Smad3 WT‐db/db and Smad3 WT‐db/m mice kidney were validated by qPCR. Db: Smad3 WT‐db/db; dm: Smad3 WT‐db/m. *P < .05 vs dm, **P < .01 vs dm. The relative expression levels of protein‐coding genes (H‐J) and lncRNAs (K‐L) in Smad3 WT‐db/db, Smad3 KO‐db/db, Smad3 WT‐db/m or Smad3 KO‐db/m mice kidney were validated by qPCR.*P < .05 vs Smad3 WT‐db/m, ***P < .001 vs Smad3 WT‐db/m, #*P* < .05 vs Smad3 KO‐db/m

It has been reported that more than 95% of pre‐mRNAs are alternatively spliced in mammals, which allows the generation of multiple splice isoforms from a single pre‐mRNA. AS process increases the coding capacity of a given gene, which can have distinct structures or functions.^51^ From the GO and KEGG results, we found that Smad3 homozygous deficiency in db/db model altered a number of protein‐coding genes related to RNA splicing. We speculated that Smad3 may affect the process of RNA splicing and then count the significant alternatively splicing events in each comparison. The distribution and number of AS events including SE, MXE, A5SS, A3SS and RI in each comparison was shown in Figure 4A. SE is the most prevalent type of AS event in all comparison, accounting for over 60% of all detected significant AS events. It is interesting that the largest number of significant AS was detected in the comparison 2 (Smad3 KO‐db/db vs Smad3 KO‐dm groups) in which the most significant GO_BP is RNA splicing and GO_CC is spliceosome complex in up‐regulated genes.

To further understand what the function of AS affected genes, we then performed GO functional enrichment and KEGG pathway analysis of differentially spliced genes (DSGs) in each comparison (Figure S2‐S5). In either Smad3 WT‐db/db or Smad3 KO‐db/db kidney, DSGs were mainly enriched in the pathway of MAPK signalling pathway. In diabetic models, Smad3 homozygous or heterozygous KO induce AS in genes participate in immune and infection pathways.

### 3.6 The co‐expression network of lncRNAs and protein‐coding genes

To explore the putative functions of lncRNAs in DKD, the co‐expression network was constructed based on the correlation analysis between the differentially expressed protein‐coding genes and lncRNAs. The Pearson's correlation coefficients were calculated for significantly correlated pairs of protein‐coding genes and lncRNAs with pcc > 0.99 or pcc > 0.9 and *P*‐value < .05 as indicated in each comparison in Table S14. Correlated pairs of protein‐coding genes and lncRNAs were chosen to build the network. Most genes interacted with few partners, whereas a small but significant proportion of them, the “hubs”, interacted with many partners.

As shown in the network analysis (Figure 4B), in comparison 1, four lncRNAs (NONMMUG000739.2, NONMMUG020039.2, NONMMUG032975.2 and NONMMUG077362.1) were identified as “hub‐node” genes with a relatively higher degree centrality. The core domain contains four lncRNAs co‐expressed with genes appear to be involved in lipid disorders and metabolic syndrome caused by obesity and type‐2 diabetes (Table S14). Lepr and NONMMUG002080.2 are the “bottleneck” genes in the network which connected several complexes or peripheral members of central complexes. In some correlated pairs, protein‐coding genes are also the closest gene of lncRNA, such as NONMMUG032975.2 and G3bp2. In comparison 2 (Figure 4C), 143 pairs of protein‐coding genes and lncRNAs were found closely correlated with threshold at pcc > 0.99 and *P*‐value < .05. The genes in the core of the network such as Gngt1 (guanine nucleotide‐binding protein, gamma transducing activity polypeptide 1), Tia1 (cytotoxic granule associated RNA‐binding protein), FUS (RNA‐binding protein), SNAPC3 (Small nuclear RNA activating complex polypeptide 3) and E2f7 (E2F transcription factor 7) play critical role in the cellular processes such as transcription regulation, RNA splicing, RNA transport and DNA repair. LncRNA NONMMUG012781.2 and NONMMUG004383.2 were found have a relatively higher co‐expression rate in the centre of network; it suggests that these lncRNAs may have similar functions. The results of co‐expression network analysis in comparisons 1 and 2 reflected gene alteration related to Smad‐KO in DKD.

In comparison 3 (Figure 4D), 982 lncRNA and protein‐coding gene pairs were found correlated. Many Olfr and vomeronasal receptor genes have higher co‐expression rate in the centre, suggested that genes may have many interaction partners related to DKD. In comparison 4 (Figure 4E), NONMMUG035375.2 was found to be a hub gene in the network centre with co‐expression to 33 protein‐coding genes. The closest protein‐coding gene of NONMMUG035375.2 is Tax1bp1 (Tax1 binding protein 1) which was found significantly decreased in diabetic mouse and regulate the biological process of autophagy and mitochondrial dysfunction.^52^, ^53^ LncRNA NONMMUG053244.1 is the hub of another separated net complex, with reaction partner of Uhrf1 (ubiquitin like with PHD and ring finger domains 1), Dtl (denticleless E3 ubiquitin protein ligase homolog), Aunip (aurora kinase A and ninein interacting protein), Hist2h2bb (histone cluster 2, H2bb) and Ercc6l (ERCC excision repair 6 like). These genes are key determinant in the biological process of mitosis, DNA damage and repair, suggesting LncRNA NONMMUG053244.1 play a role in DNA replication and repair.

### 3.7 Real‐time quantitative PCR validation of DEGs

The expression levels of seven candidate protein‐coding genes and five lncRNAs were validated by quantitative PCR (qPCR). Total RNA of kidney tissue from Smad3 WT/KO‐dm and db/db mice at 20 weeks was used for quantitative analysis. Specific primers were listed in Table S3. As shown in Figure 4F‐L, seven protein‐coding genes (Upk1b, Eda2r, Crybg2, Slitrk6, Il11ra2, Psca andGdf15) and five lncRNAs (Lnc‐Ptpn1, Lnc‐Trabd2b, Lnc‐NUS1, Lnc‐Sestd1 and Lnc‐G3bp2) were selected for qPCR validation. As shown in Table S15, although change fold was different between qPCR and RNA‐seq, the expression patterns were consistent between these two techniques.

## 4 DISCUSSION

Although the most important risk factor for DKD is hyperglycaemia, additional risk factors beyond hyperglycaemia are also involved in the process of renal injury. These include a range of metabolic factors, including excess fatty acids, carbonyl and oxidative stress.^54^ Changes in the external environment caused by diabetes can cause compensatory changes in the transcriptome of the kidney intrinsic cells which including tubular epithelial, cells podocytes, mesangial cells or glomerular endothelial cells. Pathogenetic pathways initiated and sustained in the kidney by elevated glucose levels can be enhanced by several different factors. The best known of these sensors include mammalian target of rapamycin (mTOR), 5′AMP‐activated protein kinase (AMPK) and PPAR.

TGF‐β/Smad3 signalling has been recognized as a critical pathway leading to tissue scarring as mice lacking Smad3 are protected from tissue fibrosis in a number of chronic diseases including the kidney, lung, liver and cardiac fibrosis.^55^ Moreover, Smad3 KO results in impaired mucosal immunity and diminished T cell responsiveness to TGF‐beta.^56^ In our recent study, Smad3 KO in db/db mice were found to be protected from the development of diabetic kidney injury, characterized by the normal levels of urinary albumin excretion and serum creatinine without any evidence for renal fibrosis and inflammation. However, Smad3^+/−^ show no protective effect on diabetic kidney injury.^16^ Therefore, to further investigate the alteration at the molecular level, transcriptome analysis of the kidney tissue from wildtype, Smad3 homozygous and heterozygous KO diabetic db/db mice were performed by using RNA‐seq.

Previous findings showed that hyperglycaemia accompany with elevated plasma concentrations of inflammatory molecules, such as cytokines, chemokines, adhesion molecules, growth factors and fatty acid, was found in diabetic patients, and being strong predictors of the development of diabetes.^57^ The critical metabolic changes in diabetes mean that genes might be compensated dysregulated in highly metabolic organs, such as the kidney. Results from this study confirmed that the largest numbers of DEGs which have the biological function of metabolism were altered during the development of DKD, including those genes that participate in hormone, steroid and fatty acid metabolism (shown in Figure 2 and Table S8). The change of functional genes mostly clustered in the pathway of cytokine‐cytokine receptor interaction and cellular signalling such as MAPK and PPAR signalling cascade in the diabetic kidney (shown in Figure 2 and Table S9), which are functionally connected kinases that regulate the key cellular process involved in cell survival, death, differentiation, proliferation and metabolism.^58^, ^59^

The most interesting finding of this study was Smad3 homozygous KO from db/db mice affected genes mainly involved in RNA splicing and metabolism, but heterozygosity deletion significantly altered genes related to cell division and cell cycle. Regulation of RNA splicing is a critical step of gene expression. Smad3 KO significantly increased the events of skipped exon, which directly impacted on translation of genetic information into protein. The kidney is one of the principal organs for drug and xenobiotic metabolism. The most important drug metabolism enzyme family is cytochrome P‐450, a superfamily of membrane‐bound isoenzymes that catalyses the oxidation of many drugs. Ten genes of cytochrome P‐450 superfamily (Cyp2d40, Cyp27b1, Cyp2c69, Cyp2j7, Cyp2a5, Cyp2j9, Cyp2d22, Cyp51, Cyp4a12a and Cyp7b1) were altered in Smad3 KO diabetic kidney. This implies that the protective role of Smad3 homozygous KO in DKD may relate to transcripts participating in RNA splicing and xenobiotic metabolism. The analysis of RNA‐seq data were consistent with our experimental results found in animal models.

To explore core genes and predict the function of unknown lncRNAs in the process of DKD, the co‐expression analysis of protein‐coding and lncRNAs was performed. It may help us to reveal a lot of exciting and uncovered important genes in diabetic related kidney injury. Even more importantly, it may provide a novel view on their integrative functions. It is reported that the mortality rate of knocking out the gene encoding the hub protein is three times higher than that of non‐hub in yeast.^60^ Our data also have some shortcomings, because the co‐expression network analysis of protein‐coding and lncRNAs is based on the protein interaction network database, there may be some false‐positives for prediction of non‐coding lncRNAs, which need further experimental proven.

In this study, we profiled the whole transcriptome including mRNA and ncRNA transcripts in Smad3 WT and Smad3 KO diabetic kidney by RNA‐seq. The results from our transcriptome analysis may provide novel insights into gene expression and regulatory network in diabetic kidney, which may also enable us to a better identification of the appropriate therapy targets. Because the RNA‐Seq and qPCR were performed with whole kidney samples, our results were unable to identify the regulatory role of Smad3 in cell‐type‐dependent DEGs expression during the development of DKD. Further studies need to be performed to clarify the spatial and temporal expression patterns of these DEGs within both normal and diseased kidneys by updated technology such as single cell‐RNA‐seq. Furthermore, multiple “omics”, such as the genome, proteome, epigenome, metabolomics pharmacogenomics and microbiome technologies, can also be applied to define relevant biomarkers for DKD. Personalizing treatment based on the multi‐omics approaches will be helpful in developing appropriate pharmaceutical intervention programs that can guide clinical individualized treatment.

## CONFLICT OF INTEREST

No potential conflicts of interest relevant to this article were reported.

## AUTHOR CONTRIBUTIONS

**Qin Zhou:** Conceptualization (lead); Data curation (lead); Methodology (lead); Writing‐original draft (lead). **Honghong Guo:** Formal analysis (equal); Software (equal). **Chaolun Yu:** Methodology (supporting); Resources (supporting). **Xiaoru Huang:** Conceptualization (supporting); Writing‐original draft (supporting). **Liying Liang:** Investigation (supporting); Validation (supporting). **Puhua Zhang:** Methodology (supporting); Resources (supporting); Writing‐original draft (supporting). **Jianwen Yu:** Methodology (supporting); Software (supporting). **Jizhou Zhang:** Formal analysis (supporting); Supervision (supporting); Visualization (supporting). **Ting Fung Chan:** Conceptualization (supporting); Writing‐review & editing (equal). **Ronald CW Ma:** Funding acquisition (supporting); Writing‐review & editing (supporting). **Hui‐yao Lan:** Project administration (lead); Supervision (lead).

## Supporting information

Fig S1‐S5

Table S1‐S16

## Data Availability

RNA‐seq data analysed during the current study are available via NCBI Gene Expression Omnibus (GEO) repository with the Accession No # (PRJNA675958).

## References

[1] Zhang L , Long J , Jiang W , et al. Trends in chronic kidney disease in China. N Engl J Med. 2016;375:905‐906. 27579659 10.1056/NEJMc1602469

[2] Wen L , Zhang Z , Peng R , et al. Whole transcriptome analysis of diabetic nephropathy in the db/db mouse model of type 2 diabetes. J Cell Biochem. 2019;120:17520‐17533. 31106482 10.1002/jcb.29016

[3] Salem RM , Todd JN , Sandholm N , et al. Genome‐Wide association study of diabetic kidney disease highlights biology involved in glomerular basement membrane collagen. J Am Soc Nephrol. 2019;30:2000‐2016. 31537649 10.1681/ASN.2019030218 PMC6779358

[4] Raina P , Sikka R , Kaur R , et al. Association of Transforming Growth Factor Beta‐1 (TGF‐β1) genetic variation with type 2 diabetes and end stage renal disease in two large population samples from North India. OMICS. 2015;19:306‐317. 25871499 10.1089/omi.2015.0005 PMC4424967

[5] Hameed I , Masoodi SR , Malik PA , et al. Genetic variations in key inflammatory cytokines exacerbates the risk of diabetic nephropathy by influencing the gene expression. Gene. 2018;661:51‐59. 29605608 10.1016/j.gene.2018.03.095

[6] Tayel SI , Fouda EAM , Elshayeb EI , et al. Biochemical and molecular study on interleukin‐1β gene expression and relation of single nucleotide polymorphism in promoter region with type 2 diabetes mellitus. J Cell Biochem. 2018;119:5343‐5349. 29323730 10.1002/jcb.26667

[7] Meng XM , Nikolic‐Paterson DJ , Lan HY . TGF‐beta: the master regulator of fibrosis. Nat Rev Nephrol. 2016;12:325‐338. 27108839 10.1038/nrneph.2016.48

[8] Shaker OG , Sadik NA . Transforming growth factor beta 1 and monocyte chemoattractant protein‐1 as prognostic markers of diabetic nephropathy. Hum Exp Toxicol. 2013;32:1089‐1096. 23515495 10.1177/0960327112470274

[9] Ibrahim S , Rashed L . Estimation of transforming growth factor‐beta 1 as a marker of renal injury in type II diabetes mellitus. Saudi Med J. 2007;28:519‐523. 17457470

[10] Kanwar YS , Sun L , Xie P , et al. A glimpse of various pathogenetic mechanisms of diabetic nephropathy. Annu Rev Pathol. 2011;6:395‐423. 21261520 10.1146/annurev.pathol.4.110807.092150 PMC3700379

[11] Yadav H , Quijano C , Kamaraju AK , et al. Protection from obesity and diabetes by blockade of TGF‐beta/Smad3 signaling. Cell Metab. 2011;14:67‐79. 21723505 10.1016/j.cmet.2011.04.013 PMC3169298

[12] Fain JN , Tichansky DS , Madan AK . Transforming growth factor beta1 release by human adipose tissue is enhanced in obesity. Metabolism. 2005;54:1546‐1551. 16253647 10.1016/j.metabol.2005.05.024

[13] Voelker J , Berg PH , Sheetz M , et al. Anti‐TGF‐beta1 antibody therapy in patients with diabetic nephropathy. J Am Soc Nephrol. 2017;28:953‐962. 27647855 10.1681/ASN.2015111230 PMC5328150

[14] Gu YY , Liu XS , Huang XR , et al. Diverse role of TGF‐β in kidney disease. Front Cell Dev Biol. 2020;28(8):123. 10.3389/fcell.2020.00123 PMC7093020 32258028

[15] Ma TT , Meng XM . TGF‐β/Smad and renal fibrosis. Adv Exp Med Biol. 2019;1165:347‐364. 31399973 10.1007/978-981-13-8871-2_16

[16] Xu BH , Sheng J , You YK , et al. Deletion of Smad3 prevents renal fibrosis and inflammation in type 2 diabetic nephropathy. Metabolism. 2020;103:154013. 31734275 10.1016/j.metabol.2019.154013

[17] Ashcroft GS , Yang X , Glick AB , et al. Mice lacking Smad3 show accelerated wound healing and an impaired local inflammatory response. Nat Cell Biol. 1999;1:260‐266. 10559937 10.1038/12971

[18] Chen S , Zhou Y , Chen Y , et al. fastp: an ultra‐fast all‐in‐one FASTQ preprocessor. Bioinformatics. 2018;34:i884‐i890. 30423086 10.1093/bioinformatics/bty560 PMC6129281

[19] Kim D , Langmead B , Salzberg SL . HISAT: a fast spliced aligner with low memory requirements. Nat Methods. 2015;12:357‐360. 25751142 10.1038/nmeth.3317 PMC4655817

[20] Pertea M , Pertea GM , Antonescu CM , et al. StringTie enables improved reconstruction of a transcriptome from RNA‐seq reads. Nat Biotechnol. 2015;33:290‐295. 25690850 10.1038/nbt.3122 PMC4643835

[21] Niknafs YS , Pandian B , Iyer HK , et al. TACO produces robust multisample transcriptome assemblies from RNA‐seq. Nat Methods. 2017;14:68‐70. 27869815 10.1038/nmeth.4078 PMC5199618

[22] Quinlan AR , Hall IM . BEDTools: a flexible suite of utilities for comparing genomic features. Bioinformatics. 2010;26:841‐842. 20110278 10.1093/bioinformatics/btq033 PMC2832824

[23] Wang L , Park HJ , Dasari S , et al. CPAT: Coding‐Potential Assessment Tool using an alignment‐free logistic regression model. Nucleic Acids Res. 2013;41:e74. 23335781 10.1093/nar/gkt006 PMC3616698

[24] Li A , Zhang J , Zhou Z . PLEK: a tool for predicting long non‐coding RNAs and messenger RNAs based on an improved k‐mer scheme. BMC Bioinformatics. 2014;15:311. 25239089 10.1186/1471-2105-15-311 PMC4177586

[25] Patro R , Duggal G , Love MI , et al. Salmon provides fast and bias‐aware quantification of transcript expression. Nat Methods. 2017;14:417‐419. 28263959 10.1038/nmeth.4197 PMC5600148

[26] Soneson C , Love MI , Robinson MD . Differential analyses for RNA‐seq: transcript‐level estimates improve gene‐level inferences. F1000Res. 2015;4:1521. 26925227 10.12688/f1000research.7563.1 PMC4712774

[27] Yu G , Wang LG , Han Y , et al. clusterProfiler: an R package for comparing biological themes among gene clusters. OMICS. 2012;16:284‐287. 22455463 10.1089/omi.2011.0118 PMC3339379

[28] Shen S , Park JW , Lu ZX , et al. rMATS: robust and flexible detection of differential alternative splicing from replicate RNA‐Seq data. Proc Natl Acad Sci USA. 2014;111:E5593‐E5601. 25480548 10.1073/pnas.1419161111 PMC4280593

[29] Livak KJ , Schmittgen TD . Analysis of relative gene expression data using real‐time quantitative PCR and the 2(‐Delta Delta C(T)) Method. Methods. 2001;25:402‐408. 11846609 10.1006/meth.2001.1262

[30] Rinn JL , Chang HY . Genome regulation by long noncoding RNAs. Annu Rev Biochem. 2012;81:145‐166. 22663078 10.1146/annurev-biochem-051410-092902 PMC3858397

[31] Ulitsky I , Bartel DP . lincRNAs: genomics, evolution, and mechanisms. Cell. 2013;154:26‐46. 23827673 10.1016/j.cell.2013.06.020 PMC3924787

[32] Liu RZ , Garcia E , Glubrecht DD , et al. CRABP1 is associated with a poor prognosis in breast cancer: adding to the complexity of breast cancer cell response to retinoic acid. Mol Cancer. 2015;14:129. 26142905 10.1186/s12943-015-0380-7 PMC4491424

[33] Ueda T , Sakamaki K , Kuroki T , et al. Molecular cloning and characterization of the chromosomal gene for human lactoperoxidase. Eur J Biochem. 1997;243:32‐41. 9030719 10.1111/j.1432-1033.1997.0032a.x

[34] Jiang J , Creasy KT , Purnell J , et al. Zhx2 (zinc fingers and homeoboxes 2) regulates major urinary protein gene expression in the mouse liver. J Biol Chem. 2017;292:6765‐6774. 28258223 10.1074/jbc.M116.768275 PMC5399123

[35] Zhang J , Ahn J , Suh Y , et al. Identification of CTLA2A, DEFB29, WFDC15B, SERPINA1F and MUP19 as novel tissue‐specific secretory factors in mouse. PLoS One. 2015;10:e0124962. 25946105 10.1371/journal.pone.0124962 PMC4422522

[36] Echtay KS , Roussel D , St‐Pierre J , et al. Superoxide activates mitochondrial uncoupling proteins. Nature. 2002;415:96‐99. 11780125 10.1038/415096a

[37] Kapfhamer J , Waite C , Ascoli M . The Galphaq/11‐provoked induction of Akr1c18 in murine luteal cells is mediated by phospholipase C. Mol Cell Endocrinol. 2018;470:179‐187. 29107092 10.1016/j.mce.2017.10.012

[38] Meech R , Hu DG , McKinnon RA , et al. The UDP‐Glycosyltransferase (UGT) superfamily: new members, new functions, and novel paradigms. Physiol Rev. 2019;99:1153‐1222. 30724669 10.1152/physrev.00058.2017

[39] Gburcik V , Cleasby ME , Timmons JA . Loss of neuronatin promotes "browning" of primary mouse adipocytes while reducing Glut1‐mediated glucose disposal. Am J Physiol Endocrinol Metab. 2013;304:E885‐E894. 23482445 10.1152/ajpendo.00463.2012 PMC3625784

[40] Shum EY , Espinoza JL , Ramaiah M , et al. Identification of novel post‐transcriptional features in olfactory receptor family mRNAs. Nucleic Acids Res. 2015;43:9314‐9326. 25908788 10.1093/nar/gkv324 PMC4627058

[41] Hoover KC . Evolution of olfactory receptors. Methods Mol Biol. 2013;1003:241‐249. 23585047 10.1007/978-1-62703-377-0_18

[42] Firestein S . How the olfactory system makes sense of scents. Nature. 2001;413:211‐218. 11557990 10.1038/35093026

[43] La Cava A . Leptin in inflammation and autoimmunity. Cytokine. 2017;98:51‐58. 27916613 10.1016/j.cyto.2016.10.011 PMC5453851

[44] Tanaka N , Matsubara T , Krausz KW , et al. Disruption of phospholipid and bile acid homeostasis in mice with nonalcoholic steatohepatitis. Hepatology. 2012;56:118‐129. 22290395 10.1002/hep.25630 PMC6371056

[45] Vallee Marcotte B , Guenard F , Cormier H , et al. Plasma triglyceride levels may be modulated by gene expression of IQCJ, NXPH1, PHF17 and MYB in humans. Int J Mol Sci. 2017;18. 10.3390/ijms18020257 PMC5343793 28134766

[46] Engreitz JM , Haines JE , Perez EM , et al. Local regulation of gene expression by lncRNA promoters, transcription and splicing. Nature. 2016;539:452‐455. 27783602 10.1038/nature20149 PMC6853796

[47] Carpenter AR , Becknell MB , Ching CB , et al. Uroplakin 1b is critical in urinary tract development and urothelial differentiation and homeostasis. Kidney Int. 2016;89:612‐624. 26880456 10.1016/j.kint.2015.11.017 PMC4757817

[48] Borner T , Shaulson ED , Ghidewon MY , et al. GDF15 induces anorexia through nausea and emesis. Cell Metab. 2020;31:351‐362. e355. 31928886 10.1016/j.cmet.2019.12.004 PMC7161938

[49] Kempf T , Zarbock A , Widera C , et al. GDF‐15 is an inhibitor of leukocyte integrin activation required for survival after myocardial infarction in mice. Nat Med. 2011;17:581‐588. 21516086 10.1038/nm.2354

[50] Zhao B , Li H , Liu J , et al. MicroRNA‐23b targets Ras GTPase‐Activating Protein SH3 domain‐binding protein 2 to alleviate fibrosis and albuminuria in diabetic nephropathy. J Am Soc Nephrol. 2016;27:2597‐2608. 26839366 10.1681/ASN.2015030300 PMC5004638

[51] Kornblihtt AR , Schor IE , Allo M , et al. Alternative splicing: a pivotal step between eukaryotic transcription and translation. Nat Rev Mol Cell Biol. 2013;14:153‐165. 23385723 10.1038/nrm3525

[52] Biel TG , Aryal B , Gerber MH , et al. Mitochondrial dysfunction generates aggregates that resist lysosomal degradation in human breast cancer cells. Cell Death Dis. 2020;11:460. 32541677 10.1038/s41419-020-2658-y PMC7296005

[53] Xiao Y , Wu QQ , Duan MX , et al. TAX1BP1 overexpression attenuates cardiac dysfunction and remodeling in STZ‐induced diabetic cardiomyopathy in mice by regulating autophagy. Biochim Biophys Acta Mol Basis Dis. 2018;1864:1728‐1743. 29476905 10.1016/j.bbadis.2018.02.012

[54] Thomas MC , Brownlee M , Susztak K , et al. Diabetic kidney disease. Nat Rev Dis Primers. 2015;1:15018. 27188921 10.1038/nrdp.2015.18 PMC7724636

[55] Huang XR , Chung AC , Yang F , et al. Smad3 mediates cardiac inflammation and fibrosis in angiotensin II‐induced hypertensive cardiac remodeling. Hypertension. 2010;55:1165‐1171. 20231525 10.1161/HYPERTENSIONAHA.109.147611

[56] Yang X , Letterio JJ , Lechleider RJ , et al. Targeted disruption of SMAD3 results in impaired mucosal immunity and diminished T cell responsiveness to TGF‐beta. EMBO J. 1999;18:1280‐1291. 10064594 10.1093/emboj/18.5.1280 PMC1171218

[57] Donate‐Correa J , Martin‐Nunez E , Muros‐de‐Fuentes M , et al. Inflammatory cytokines in diabetic nephropathy. J Diabetes Res. 2015;2015:948417. 25785280 10.1155/2015/948417 PMC4345080

[58] Ma FY , Tesch GH , Ozols E , et al. TGF‐beta1‐activated kinase‐1 regulates inflammation and fibrosis in the obstructed kidney. Am J Physiol Renal Physiol. 2011;300:F1410‐F1421. 21367917 10.1152/ajprenal.00018.2011

[59] Zhou J , Fan Y , Zhong J , et al. TAK1 mediates excessive autophagy via p38 and ERK in cisplatin‐induced acute kidney injury. J Cell Mol Med. 2018;22:2908‐2921. 29504713 10.1111/jcmm.13585 PMC5908118

[60] Han JD , Bertin N , Hao T , et al. Evidence for dynamically organized modularity in the yeast protein‐protein interaction network. Nature. 2004;430:88‐93. 15190252 10.1038/nature02555

