# Supplementary material for: Identification of Smad3‐related transcriptomes in type‐2 diabetic nephropathy by whole transcriptome RNA sequencing
Source: J Cell Mol Med. 2020 Dec 25;25(4):2052–68. doi: 10.1111/jcmm.16133 (PMC7882931; doi:10.1111/jcmm.16133)

# Supplementary Figure 1

## Correlation of gene expression among all samples

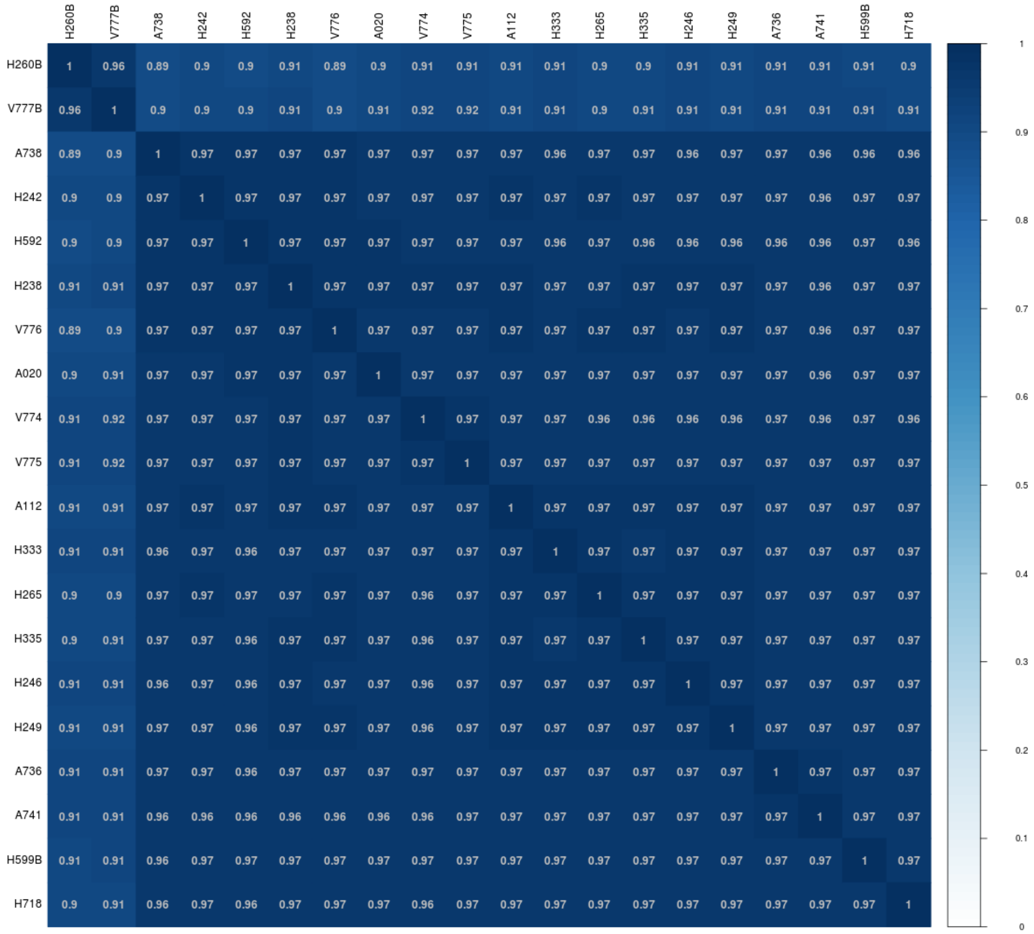

# Supplementary Figure 2

## GO analysis of DSGs in comparison 1

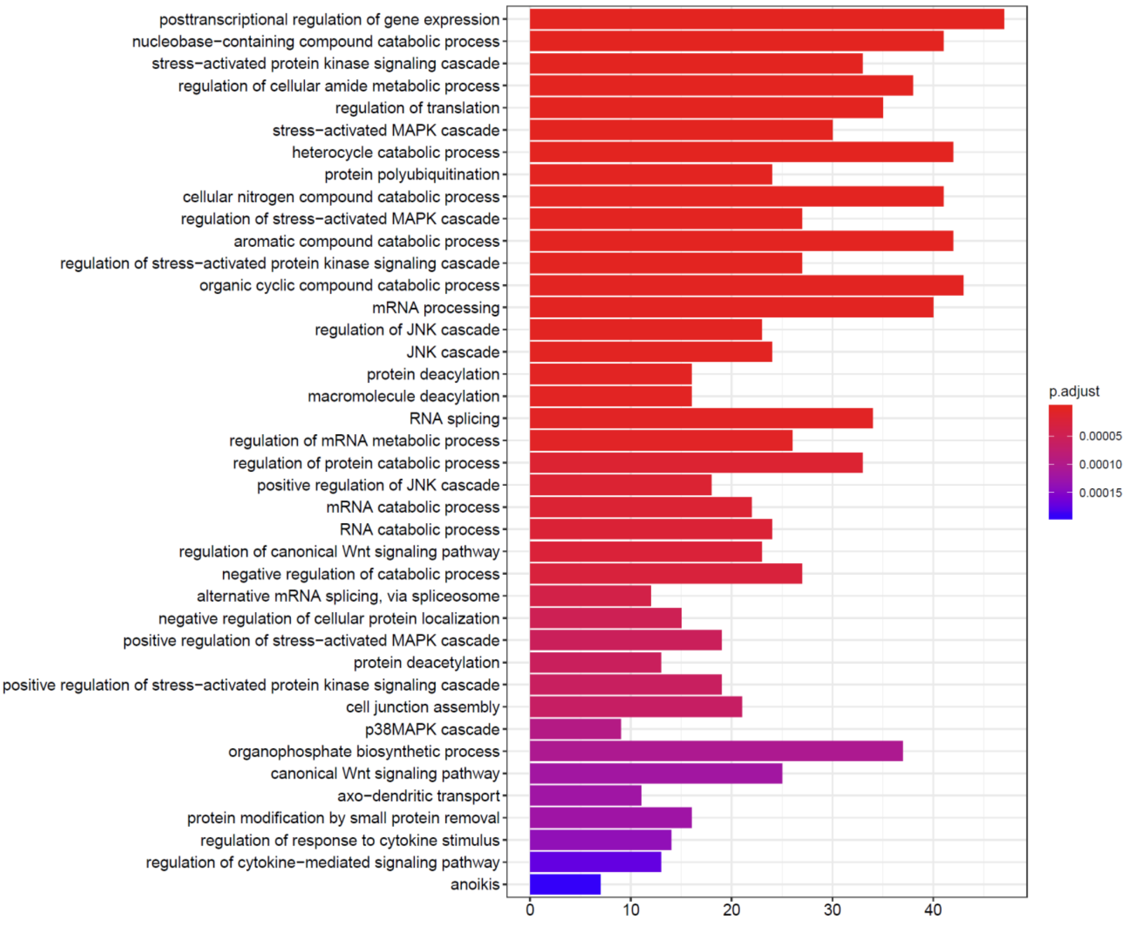


## KEGG pathway analysis of DSGs in comparison 1

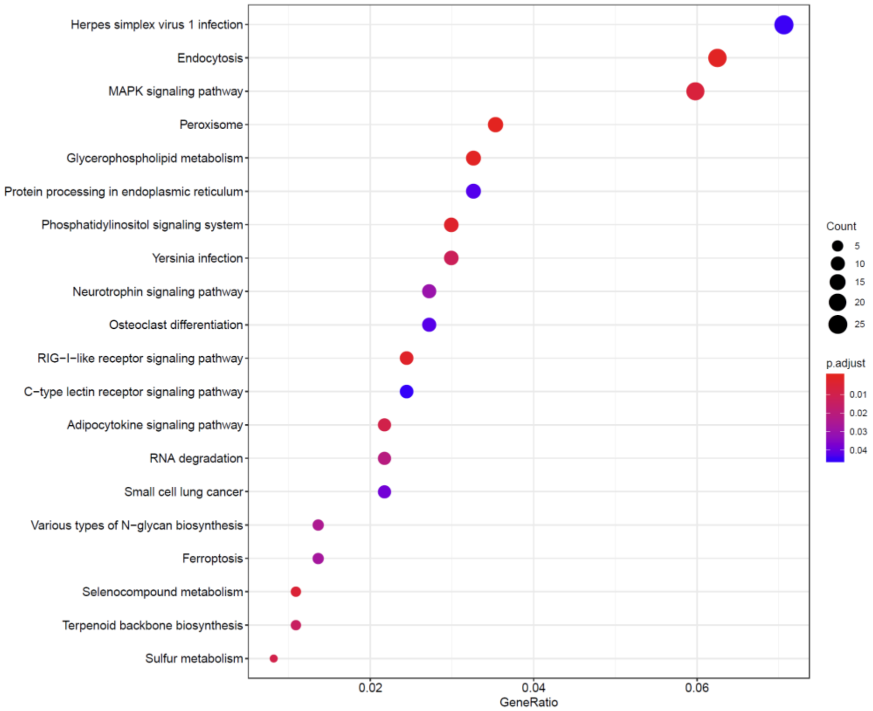

Supplementary Figure 3

GO analysis of DSGs in comparison 2

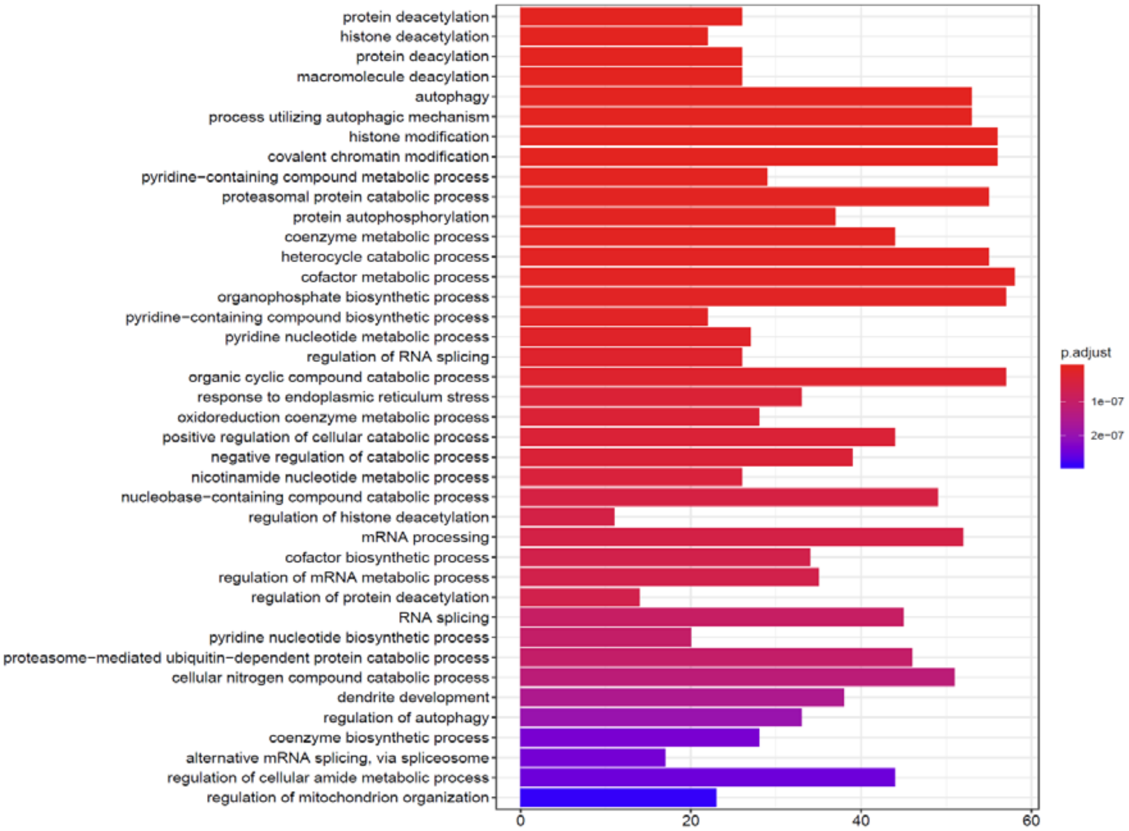


KEGG pathway analysis of DSGs in comparison 2

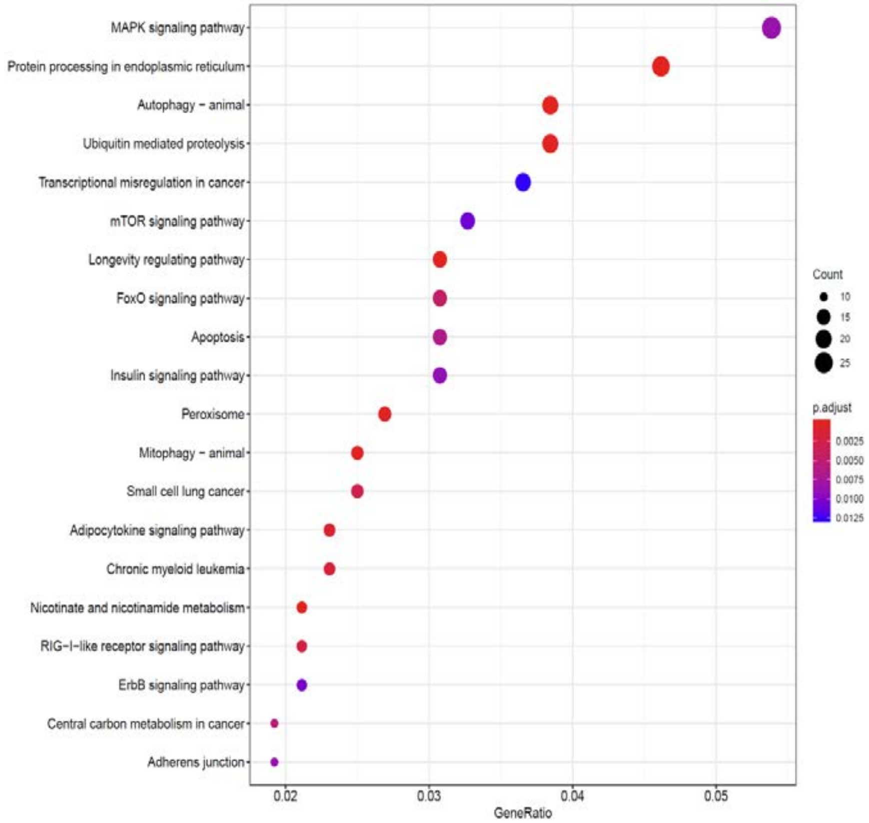

# Supplementary Figure 4

## GO analysis of DSGs in comparison 3

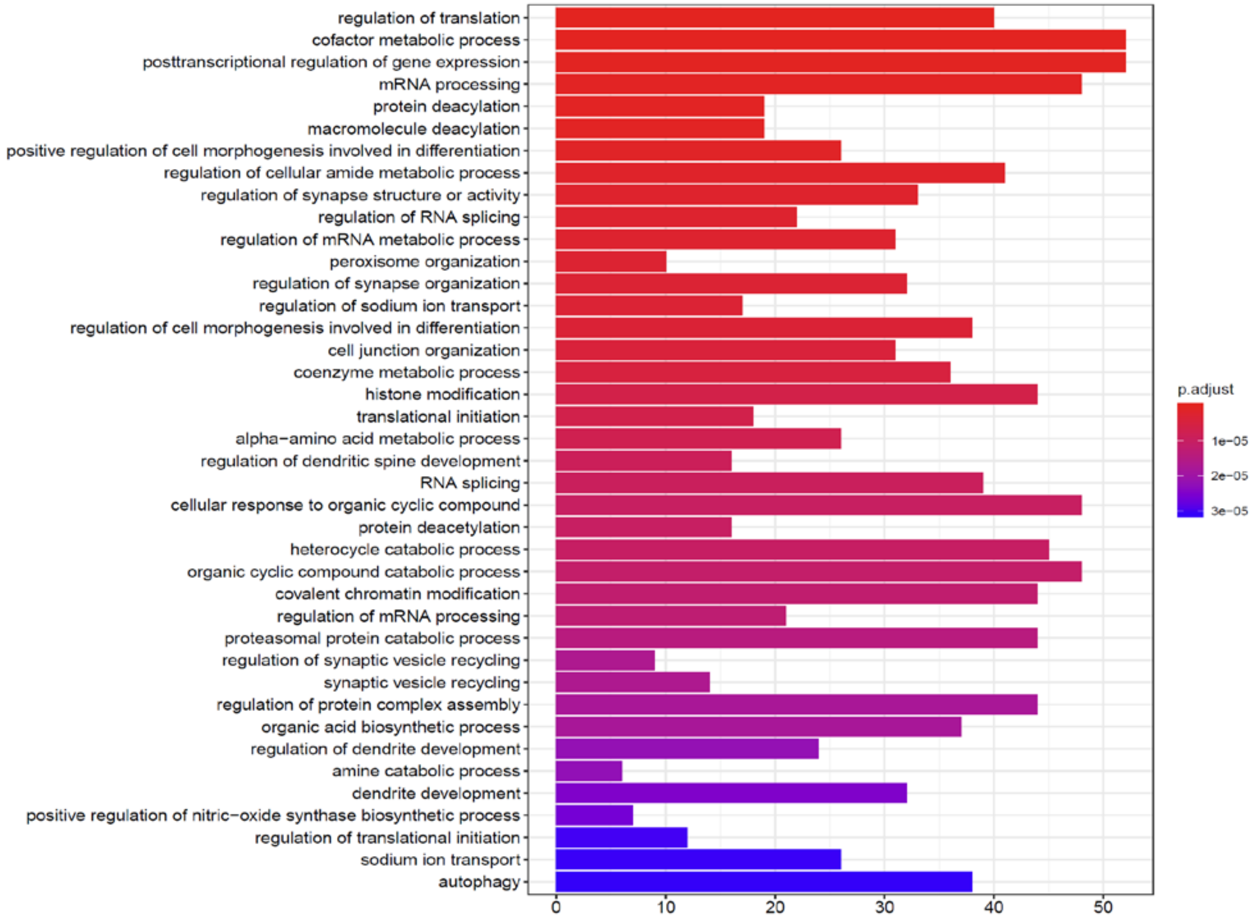


## KEGG pathway analysis of DSGs in comparison 3

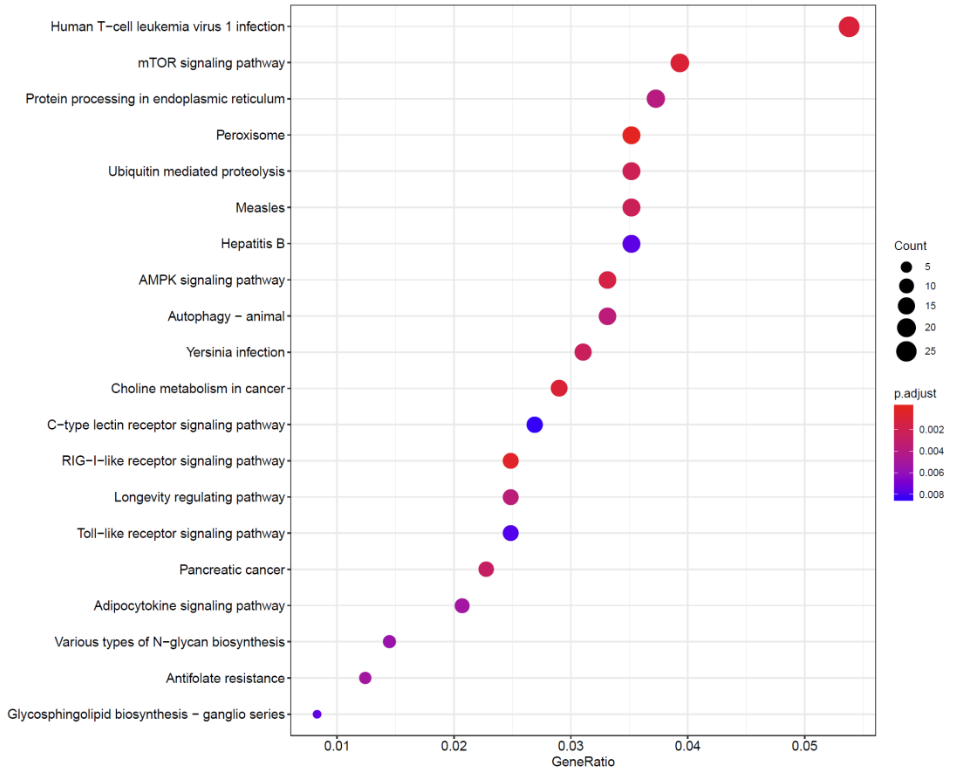

Supplementary Figure 5

## GO analysis of DSGs in comparison 4

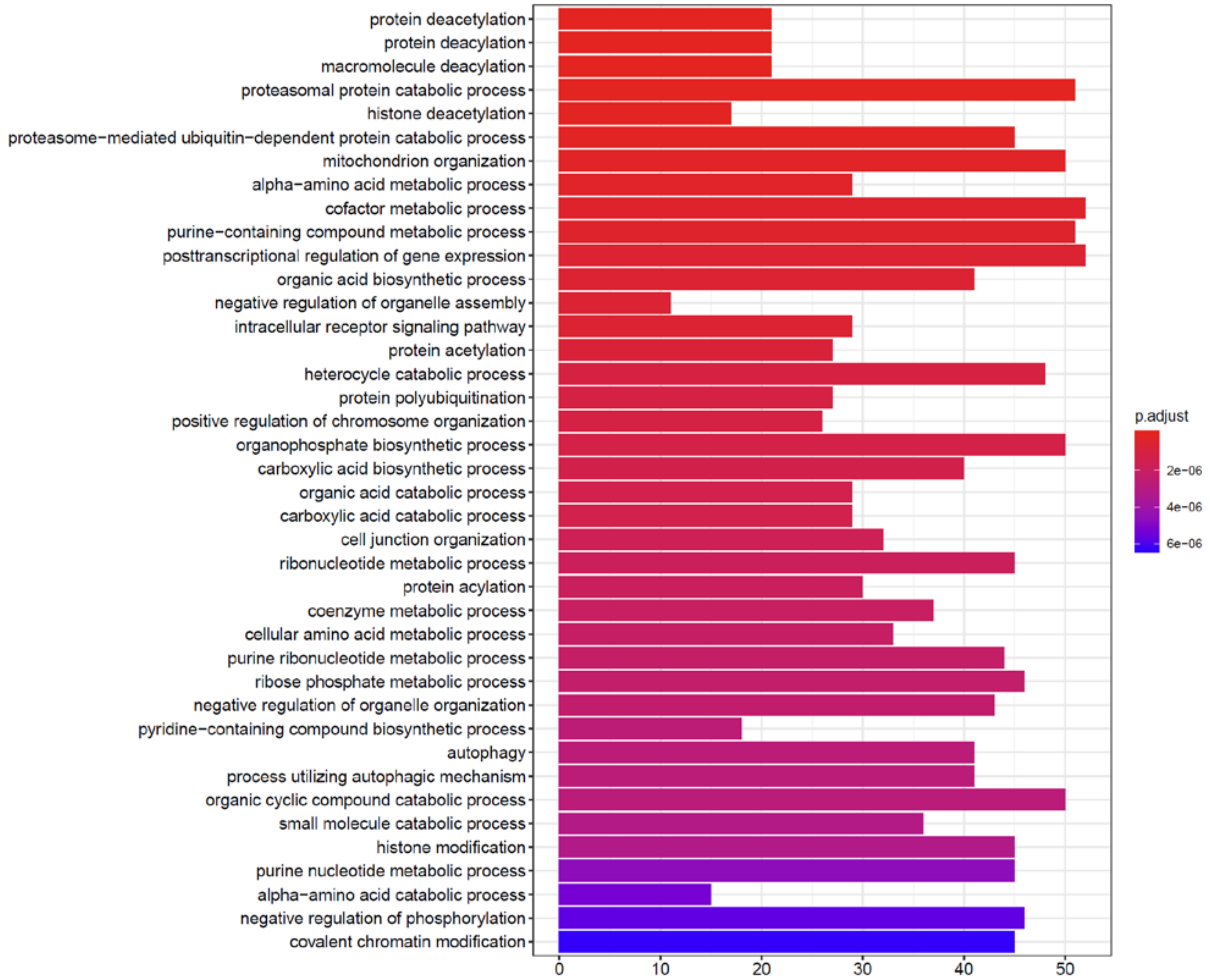


## KEGG pathway analysis of DSGs in comparison 4

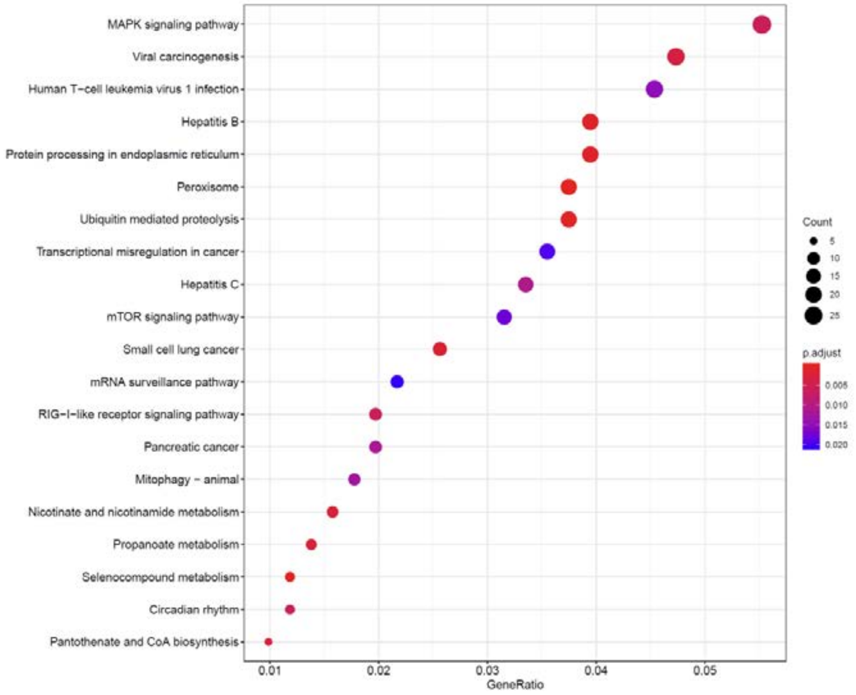

Supplement: Supplementary file 1 — Fig S1‐S5 [file JCMM-25-2052-s001.pdf]
